# Neutrophil extracellular trap biomarkers in aneurysmal subarachnoid hemorrhage: early decline of DNase 1 activity associated with delayed cerebral ischemia

**DOI:** 10.3389/fneur.2024.1354224

**Published:** 2024-04-19

**Authors:** Philipp Hendrix, Jens Witsch, Valérie Spalart, Hauke Schneider, Joachim Oertel, Jürgen Geisel, Kimberly Martinod, Sina Hemmer

**Affiliations:** ^1^Department of Neurosurgery, Saarland University Medical Center, Homburg, Germany; ^2^Department of Neurosurgery, Geisinger Medical Center, Danville, PA, United States; ^3^Department of Neurology, Perelman School of Medicine, University of Pennsylvania, Philadelphia, PA, United States; ^4^Center for Molecular and Vascular Biology, Department of Cardiovascular Sciences, KU Leuven, Leuven, Belgium; ^5^Department of Neurology, University Hospital Augsburg, Augsburg, Germany; ^6^Department of Clinical Chemistry and Laboratory Medicine, Saarland University Medical Center, Homburg, Germany

**Keywords:** subarachnoid hemorrhage, intracranial aneurysm, neutrophil extracellular traps, cell-free DNA, DNase activity

## Abstract

**Introduction:**

High-mobility group box 1 (HMGB1) protein is a critical mediator of neutrophil extracellular trap (NET) formation (NETosis). Myeloperoxidase (MPO)-DNA complexes, a biomarker of NETs, and HMGB1 have been associated with delayed cerebral ischemia (DCI) after aneurysmal subarachnoid hemorrhage (aSAH). Additional mechanistic NET-related biomarkers and their role in the neuroinflammatory cascade surrounding DCI remain to be explored.

**Methods:**

A post-hoc analysis of a prospective, blinded, single-center biomarker observational study was performed. *De novo* measurements of serum citrullinated histone H3-DNA complexes (H3Cit-DNA), peptidylarginine deiminase 4 (PAD4), cell-free DNA (cf-DNA), and DNase 1 activity were conducted on admission (D0) and day 4 (D4). Delayed cerebral infarction (DCI) was defined as new cerebral infarction on CT head not present on the post-treatment scan.

**Results:**

H3Cit-DNA, PAD4, cf-DNA, and DNase 1 activity were within quantifiable ranges in all serum samples analyzed at D0 and D4. Admission biomarker levels were not associated with DCI development. From D0 to D4, in both the DCI and the non-DCI groups, H3Cit-DNA levels significantly decreased, cf-DNA levels significantly increased, and PAD4 levels remained stable. In contrast, DNase 1 activity significantly decreased from D0 to D4 in the DCI group (*p* < 0.001) but not in the non-DCI group.

**Conclusion:**

This exploratory analysis demonstrated NET-related biomarkers such as H3Cit-DNA, PAD4, cf-DNA, and DNase 1 activity in all aSAH patients. A decline of systemic DNase 1 activity in the early phase might increase the risk of delayed cerebral ischemia.

## Introduction

Neuroinflammation plays a key role in the pathophysiology cascades after aneurysmal subarachnoid hemorrhage (aSAH) but remains poorly understood in the context of aSAH sequelae (1–3). Hence, neuroprotective treatment options to mitigate disease burden are still limited. Platelets are increasingly recognized for their role in inflammatory processes. Their interplay with the coagulation system and the innate immune system is referred to as immunothrombosis and thromboinflammation, signifying their dual role (4, 5). Platelet-derived release of HMGB1 stimulates neutrophils to release reticulated chromatin structures termed neutrophil extracellular traps (NETs). NETs can entrap pathogens in the bloodstream, thereby contributing to host defense. However, neutrophil overstimulation causes excessive NET release, which initiates and promotes thrombus formation on the arterial and venous vascular beds (4, 6, 7). Recently, we conducted a prospective, blinded, observational single-center biomarker study to investigate the role of HMGB1 in aSAH (HIMOBASH cohort). We identified admission HMGB1 serum levels in aneurysmal subarachnoid hemorrhage patients to be associated with the risk of developing new infarctions on the CT head. We conducted a post-hoc analysis with *de novo* investigation of myeloperoxidase (MPO)-DNA complexes, a biomarker of released NETs in circulation. Effectively, for the first time, it was demonstrated that MPO-DNA complex levels were significantly associated with DCI (8, 9). These observations in context with the current literature incited us to further explore NET and NET-related biomarkers in the same cohort that are complementary to HMGB1 and MPO-DNA complexes. Here, we conducted a secondary post-hoc analysis of the HIMOBASH cohort with *de novo* serum measurements of serum citrullinated histone H3-DNA complexes (H3Cit-DNA), peptidylarginine deiminase 4 (PAD4), cell-free DNA (cf-DNA), and the measurement of DNase 1 activity as a potential regulator of serum NET levels.

## Methods

The Saarland Medical Association ethics committee approved the study (118/17). All participants or their legal representatives provided written informed consent. In HIMOBASH, consecutive patients with spontaneous, non-traumatic subarachnoid hemorrhage on CT head with suspected aneurysmal etiology were enrolled between 07/2018 and 09/2020. In 83/100 patients, aneurysmal subarachnoid hemorrhage was confirmed by digital subtraction angiography (DSA) or CT angiography (CTA). In 13/100 patients, initial DSA and repeat DSA 7–10 days after ictus were angio-negative. In 4/100 patients, DSA or CTA was not performed, and patients deceased <24 h. As previously reported (8, 9), all patients were treated according to contemporary guidelines and institutional protocol. Briefly, acute treatment was performed in a dedicated neurointensive care unit, including monitoring, acute hydrocephalus treatment via external ventricular drain (EVD) placement, endovascular or surgical aneurysm treatment within 24 h of admission, oral nimodipine administration, and bedside transcranial Doppler surveillance twice a day and additionally as required. After an aneurysm treatment, a post-treatment scan on day 1 was obtained. Delayed ischemic neurological deficits (DIND) were treated by increasing the mean arterial blood pressure using vasopressors. Decision for endovascular vasospasm treatment was made on a case-by-case basis reaching consensus between the on-call neurosurgery and neuroradiology team. Patients who failed EVD weaning underwent ventriculoperitoneal shunting. HIMOBASH outcome variable definition followed the proposed aSAH outcomes measures (10). New cerebral infarction on CT not present on the day was defined as DCI. A composite of DIND and/or pathological TCDs was defined as clinical vasospasm (CVS).

### Laboratory analyses

Serum samples were thawed at room temperature and analyzed for levels of citrullinated histone H3-DNA complexes (H3Cit-DNA), peptidylarginine deiminase 4 (PAD4), and cell-free DNA (cf-DNA). H3Cit-DNA complexes were measured using a sandwich ELISA protocol modified from Thålin et al. with capture antibody anti-histone H3 citrulline R8 (Abcam, ab232939, 1.25 μg/mL) and detection antibody peroxidase-conjugated mouse anti-DNA monoclonal antibody (Cell Death Detection ELISA^PLUS^, Roche, 11,774,425,001, 1:50 dilution) (11). PAD4 levels were quantified according to the manufacturer’s instructions with the PAD4 (human) ELISA Kit (Cayman Chemical, 501,460). cf-DNA levels were estimated from standard curves generated with the Quant-iTTM Picogreen™ dsDNA Assay (Invitrogen, P7589).

### Measurement of DNase 1 activity

DNase 1 activity was measured in the serum samples using the fluorometric DNase 1 Assay Kit from Abcam (ab234056), which quantifies DNase 1 activity based on cleavage of an exogenous DNA probe emitting a fluorescent signal. After diluting serum samples 1:2 in double-distilled water, a labeled DNA probe, reconstituted in resuspension buffer, was added to the serum samples (final dilution of the samples 1:4). DNase 1 activity was immediately measured every 30 s for 90 min at 37°C using a fluorescent plate reader (Cytation 5FV, excitation 620/40 ms, and emission 680/30 ms). All DNase activity values were normalized to the DNase activity values of the human recovered citrate (3.2%) plasma pool (Tebu-Bio, 088SER-PLP50-CUSNaC).

### Statistical analysis

Variables are displayed as frequency and percentages and median and interquartile ranges (IQR), respectively. Chi-square, Fisher’s exact, and Mann–Whitney U-tests were performed as appropriate. For paired analyses, the Wilcoxon signed-rank test was performed and Cohen’s effect sizes were calculated. *p*-values of <0.05 were considered statistically significant. SPSSv25 (IBM Corp., Armonk, NY) and GraphPad Prism 8 (San Diego, CA) were used for statistical analysis and graphical presentation.

## Results

### Study sample

A follow-up scan to determine the presence or absence of DCI was available in 78 aSAH patients. In two patients, *de novo* measurement samples could not be performed due to a lack of sufficient blood samples. Eventually, 76 aSAH patients were eligible for analysis of whom 36.8% (28/76) developed new cerebral infarction on the CT head (i.e., DCI). Baseline demographics of DCI and non-DCI patients are shown in Table 1. In patients who developed DCI compared to those who did not, median Hunt and Hess scale grades and modified Fisher scores were numerically higher (*p* = 0.083 and *p* = 0.057, respectively). Radiological and clinical vasospasm was more frequent in the DCI compared to the non-DCI group (67.9% vs. 20.8%, *p* < 0.001, and 60.7% vs. 25.0%, *p* = 0.002, respectively).

**Table 1:** Demographics in DCI vs. no-DCI patients.

Variable	DCI (*n* = 28)	Ø DCI (*n* = 48)	*p*-value
Age (IQR)	60 (46–64)	57 (51–66)	0.788
Female	15 (53.6%)	26 (54.2%)	0.960
Anterior circulation	24 (85.7%)	40 (83.3%)	1.000
Multiple aneurysms (≥ 2)	6 (21.4%)	14 (29.2%)	0.592
Arterial hypertension	15 (53.6%)	25 (52.1%)	0.900
Smoking (ever)	15 (53.6%)	23 (47.9%)	0.634
Ischemic vascular disease	3 (10.7%)	5 (10.4%)	1.000
Prior stroke	3 (10.7%)	4 (8.3%)	0.704
WFNS scale (median, IQR)	3 (1–5)	2 (1–4)	0.129
WFNS III/IV/V	14 (50.0%)	19 (39.6%)	0.377
Hunt and Hess grade (median, IQR)	3 (2–5)	3 (2–5)	0.083
Hunt and Hess IV/V	12 (42.9%)	13 (27.1%)	0.158
Modified Fisher scale (median, IQR)	4 (3–4)	3 (3–4)	0.057
modified Fisher scale III/IV	26 (92.9%)	38 (79.2%)	0.192
Intraventricular hemorrhage	21 (75.0%)	28 (58.3%)	0.143
Intracerebral hemorrhage	6 (21.4%)	9 (18.8%)	0.777
Hydrocephalus admission	22 (78.6%)	31 (64.6%)	0.200
Aneurysm treatment via clipping	10 (35.7%)	21 (43.8%)	0.492
Cerebral edema admission	10 (35.7%)	2 (4.2%)	0.351
Cerebral infarction day 1	13 (46.4%)	18 (37.5%)	0.445
Radiological vasospasm (CTA and DSA)	19 (67.9%)	10 (20.8%)	**<0.001**
Clinical vasospasm (DIND and TCD)	17 (60.7%)	12 (25.0%)	**0.002**
Citrullinated histone H3-DNA complexes day 0 (IQR)	528 (394–749)	701 (480–954)	0.053
Citrullinated histone H3-DNA complexes day 4^*&^ (IQR)	434 (230–605)	475 (328–754)	0.219
Peptidylarginine deiminase day 0 (IQR)	7.8 (3.3–16.9)	4.9 (1.9–10.0)	0.086
Peptidylarginine deiminase day 4^†&^ (IQR)	9.1 (3.8–13.4)	7.0 (4.7–15.1)	0.605
Cell-free DNA day 0 (IQR)	793 (618–1,090)	752 (585–908)	0.371
Cell-free DNA day 4^*§^ (IQR)	1,266 (1064–1816)	1,284 (885–1,513)	0.307
DNase 1 activity day 0 (IQR)	52.1 (40.7–62.1)	45.9 (41.3–79.1)	1.000
DNase 1 activity day 4^†&^ (IQR)	42.7 (35.5–54.7)	48.1 (36.4–75.6)	0.286

Data not available in 1 (*) and 2 (†) DCI patients, and 6 (&) and 7 (§) non-DCI patients, respectively.

### NET biomarker and DNase 1 activity levels at D0 and D4

On admission, H3Cit-DNAH3Cit-DNA, PAD4, cf-DNA, and DNase 1 activity did not show significant differences between DCI and non-DCI patients. On day 4, H3Cit-DNA, PAD4, cf-DNA, and DNase 1 activity were similar between DCI and non-DCI patients. Overall, case fatality accounted for the DCI and non-DCI cohorts to be decreased by one and six cases for the day 4 analysis (Table 1).

### Pairwise comparison of NET biomarker and DNase 1 activity levels D0 vs. D4

**Figure 1:**
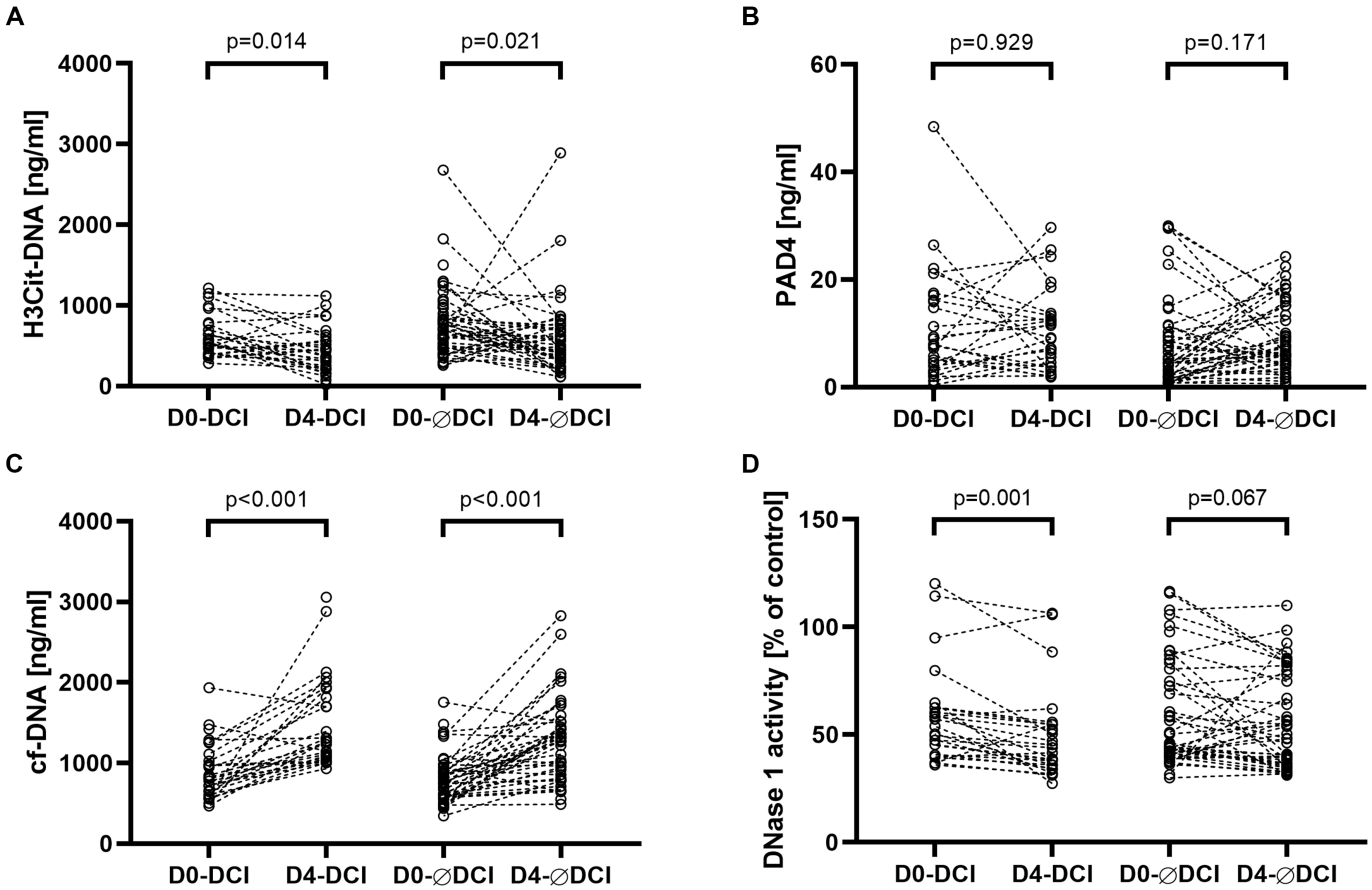
NET biomarkers H3Cit-DNA **(A)** and PAD4 **(B)** and NET-related biomarkers cf-DNA **(C)** and DNase 1 activity **(D)** are displayed in separate panels. Each panel compares biomarker levels of subgroups who suffered DCI on admission (D0-DCI) and day 4 (D4-DCI) and did not suffer DCI on admission (D0-ØDCI) and day 4 (D4-ØDCI). Within the DCI and non-DCI (ØDCI) groups, a pairwise comparison was performed between admission day and day four.

Among all aSAH patients, H3Cit-DNA levels significantly decreased from day 0 to day 4 (*p* = 0.001). In the paired analysis, a significant decrease was observed in the DCI as well as the non-DCI subgroups (*p* = 0.014, *r* = −0.33 and *p* = 0.021, *r* = −0.25, respectively; Figure 1). PAD4 levels remained stable between day 0 and day 4 among all patients (*p* = 0.293). This was observed for both the DCI and non-DCI groups (*p* = 0.929 and *p* = 0.171, respectively). Cf-DNA levels significantly increased among all aSAH patients (*p* < 0.001) and the DCI vs. non-DCI groups (*p* < 0.001, *r* = 0.57 and *p* < 0.001, *r* = 0.54, respectively). Among all aSAH patients, DNase activity levels significantly decreased from day 0 to day 4 (*p* = 0.001). Of note, this decline was significant in the DCI group (*p* = 0.001, *r* = 0.47 medium effect size) but not in the non-DCI group (*p* = 0.067; Figure 1).

## Discussion

This exploratory study demonstrated that the NET biomarkers H3Cit-DNA and PAD4 as well as NET-related biomarkers cf-DNA, and DNase 1 activity could be quantified in blood samples from aSAH patients. These biomarkers have a heteromorphic correlation and show different dynamics in the early phase after aSAH.

Delayed cerebral ischemia (DCI) and early brain injury are the predominant drivers of poor outcomes and high case fatality after aneurysmal subarachnoid hemorrhage (aSAH) (12–14). Various pathomechanisms have been identified to initiate, promote, or contribute to DCI as well as EBI following cerebral aneurysm rupture. The occurrence of DCI in the absence of relevant macrovascular vasospasm as well as the non-success of aggressive vasospasm management to prevent secondary infarction in many patients has shifted the focus toward mitigating the neuroinflammatory cascades (15). Cerebral aneurysm rupture and aSAH cause eucaryotic cells to passively release a plethora of damage-associated molecular patterns (DAMPs). These DAMPs are recognized by the innate immune system and promote *sterile inflammation.* High-mobility group box 1 (HMGB1) is a prototypical DAMP (16, 17). Platelets can actively release HMGB1 and thus stimulate neutrophils. Activated neutrophils release NETs that promote intravascular scaffolds of chromatin, protein, and blood cells resulting in perivascular inflammation and (micro-)thrombosis (6). The mechanisms of NET formation are not yet fully understood. Reactive oxygen species are involved in the release of myeloperoxidase (MPO) and neutrophil elastase, whereas PAD4 catalyzes the citrullination of histones H3 and H4. These processes facilitate chromatin decondensation, nucleus swelling, and ultimately nuclear membrane loss (7).

In our previous study, we observed that MPO-DNA complexes significantly decreased from day 0 to day 4 and hypothesized that thrombus-bound complexes evade serum level detection (8). Here, H3Cit-DNA levels also significantly decreased. In contrast, PAD4 activity remained stable in the first 4 days. This might indicate that PAD4-catalyzed NET formation is ongoing while MPO-DNA and H3Cit-DNA are removed from the serum by thrombus formation or thrombus binding. However, it yet remains to be determined why MPO-DNA decrease was associated with DCI but H3Cit-DNA decrease did not.

Serum cf-DNA are fragments of extracellular DNA (18). In the setting of aSAH, cf-DNA concentrations base on the extent of primary tissue injury suffered from cerebral aneurysm rupture and secondary injury from processes summarized as early brain injury and the neuroinflammatory response status. This could explain the observed significant increase in cf-DNA from admission to day 4 in all aSAH patients. Under healthy conditions, there is homeostasis of cf-DNA and their degradation via deoxyribunocleases (DNases) (18). Decreased DNase 1 activity not capable of dismantling NETs has been linked to progressive lupus nephritis (19). Additionally, the interplay of cf-DNA and DNase activity was found to be associated with the clinical course and sequelae of large vessel occlusion, stroke, and ischemic stroke (20). In an experimental SAH model, Hao *et al.* demonstrated a strong correlation between NETs and cerebral microthrombosis in the first 24 h after experimental subarachnoid hemorrhage SAH. Infusion of DNase 1 significantly inhibited NETosis, microthrombosis, brain edema, neuronal injury, and blood–brain barrier disruption (21). Similarly, in a murine SAH model, Zeng *et al.* found that exogenous DNase 1 administration mitigated NET-induced inflammatory processes (22). In our study, we observed a significant decline of DNase 1 activity from admission to day 4 in those patients who developed DCI but not in those who did not develop DCI. The risk of DCI development in these patients might be explained by a disequilibrium of NET formation to NET degradation eventually increasing the risk of microthrombosis and with that secondary infarction. The role of HMGB1 in the interplay of cf-DNA and DNase activity remains to be explored.

### Limitations and strengths

This cohort is limited to early-phase NET and NET-related biomarker assessment and complementary DNase activity measurement. Additional individual laboratory assessments potentially display dynamics not portrayed here. This cohort was not biased by not including poor-grade patients and overall represented aSAH-specific event variables comparable to large-scale aSAH cohorts. As the first of its kind in human aSAH, it acts as a framework for future biomarker study designs and may prompt additional bench research.

## Conclusion

This exploratory analysis measured NET and NET-related biomarkers, such as H3Cit-DNA, PAD4, cf-DNA, and DNase 1 activity, in a cohort of aSAH patients. A decline of DNase 1 activity in the early phase might increase the risk of delayed cerebral ischemia. This could be indicative of a disequilibrium of NET formation and NET degradation. Pharmacological targeting of this disequilibrium might attenuate aSAH sequelae such as DCI.

## Author’s note

Parts of this work have been presented at the 20^th^ Annual Meeting of the Society of NeuroInterventional Surgery (SNIS, San Diego/CA, July 31 – August 4, 2023, San Diego/CA); the 74^th^ Annual Congress of the German Society of Neurosurgery (DGNC, Stuttgart, June 25 – 28, 2023) and Arbeitstagung NeuroIntensiv Medizin (ANIM, Berlin, January 19 – 21, 2023).

## Data availability statement

The datasets presented in this article are not readily available because data are available upon reasonable request; initiation requires contacting the corresponding author (PH). Requests to access the datasets should be directed to hendrix.philipp@gmail.com.

## Ethics statement

The studies involving humans were approved by Ethics committee of the Saarland Medical Association 118/17. The studies were conducted in accordance with the local legislation and institutional requirements. The patients or their legal representatives provided informed consent.

## Author contributions

PH: Conceptualization, Data curation, Formal analysis, Funding acquisition, Investigation, Methodology, Project administration, Resources, Supervision, Validation, Visualization, Writing – original draft, Writing – review & editing. JW: Conceptualization, Methodology, Project administration, Supervision, Writing – review & editing. VS: Data curation, Investigation, Methodology, Validation, Writing – review & editing. HS: Writing – review & editing. JO: Project administration, Resources, Supervision, Writing – review & editing. JG: Formal analysis, Investigation, Methodology, Validation, Writing – review & editing. KM: Conceptualization, Data curation, Formal analysis, Investigation, Methodology, Project administration, Resources, Supervision, Validation, Writing – review & editing. SH: Conceptualization, Data curation, Formal analysis, Investigation, Methodology, Project administration, Supervision, Validation, Writing – review & editing.

## References

[1] de Oliveira Manoel AL Macdonald RL. Neuroinflammation as a Target for Intervention in Subarachnoid Hemorrhage. Front Neurol. (2018) 9:292. doi: 10.3389/fneur.2018.00292, PMID: 29770118 PMC5941982

[2] Cahill J Cahill WJ Calvert JW Calvert JH Zhang JH. Mechanisms of early brain injury after subarachnoid hemorrhage. J Cereb Blood Flow Metab. (2006) 26:1341–53. doi: 10.1038/sj.jcbfm.9600283 16482081

[3] Macdonald RL. Delayed neurological deterioration after subarachnoid haemorrhage. Nat Rev Neurol. (2014) 10:44–58. doi: 10.1038/nrneurol.2013.246 24323051

[4] Martinod K Deppermann C. Immunothrombosis and thromboinflammation in host defense and disease. Platelets. (2021) 32:314–24. doi: 10.1080/09537104.2020.1817360, PMID: 32896192

[5] Engelmann B Massberg S. Thrombosis as an intravascular effector of innate immunity. Nat Rev Immunol. (2013) 13:34–45. doi: 10.1038/nri3345, PMID: 23222502

[6] Martinod K Wagner DD. Thrombosis: tangled up in NETs. Blood. (2014) 123:2768–76. doi: 10.1182/blood-2013-10-463646, PMID: 24366358 PMC4007606

[7] Laridan E Martinod K De Meyer SF. Neutrophil Extracellular Traps in Arterial and Venous Thrombosis. Semin Thromb Hemost. (2019) 45:086–93. doi: 10.1055/s-0038-1677040 30634198

[8] Witsch J Spalart V Martinod K Schneider H Oertel J Geisel J. Neutrophil Extracellular Traps and Delayed Cerebral Ischemia in Aneurysmal Subarachnoid Hemorrhage. Critical Care Explorations. (2022) 4:e0692. doi: 10.1097/CCE.0000000000000692, PMID: 35620772 PMC9116951

[9] Hemmer S Senger S Griessenauer CJ Simgen A Oertel J Geisel J. Admission serum high mobility group box 1 (HMGB1) protein predicts delayed cerebral ischemia following aneurysmal subarachnoid hemorrhage. Neurosurg Rev. (2021) 45:807–17. doi: 10.1007/s10143-021-01607-0 34302233

[10] Vergouwen MDI Vermeulen M van Gijn J Rinkel GJE Wijdicks EF Muizelaar JP. Definition of delayed cerebral ischemia after aneurysmal subarachnoid hemorrhage as an outcome event in clinical trials and observational studies: proposal of a multidisciplinary research group. Stroke. (2010) 41:2391–5. doi: 10.1161/STROKEAHA.110.589275, PMID: 20798370

[11] Thålin C Aguilera K Hall NW Marunde MR Burg JM Rosell A. Quantification of citrullinated histones: Development of an improved assay to reliably quantify nucleosomal H3Cit in human plasma. J Thromb Haemost. (2020) 18:2732–43. doi: 10.1111/jth.15003, PMID: 32654410 PMC8722705

[12] Lauzier DC Jayaraman K Yuan JY Diwan D Vellimana AK Osbun JW. Early Brain Injury After Subarachnoid Hemorrhage: Incidence and Mechanisms. Stroke. (2023) 54:1426–40. doi: 10.1161/STROKEAHA.122.040072, PMID: 36866673 PMC10243167

[13] Dhar R Diringer MN. The burden of the systemic inflammatory response predicts vasospasm and outcome after subarachnoid hemorrhage. Neurocrit Care. (2008) 8:404–12. doi: 10.1007/s12028-008-9054-2, PMID: 18196475 PMC2538678

[14] de Rooij NK Rinkel GJE Dankbaar JW Frijns CJM. Delayed cerebral ischemia after subarachnoid hemorrhage: a systematic review of clinical, laboratory, and radiological predictors. Stroke. (2013) 44:43–54. doi: 10.1161/STROKEAHA.112.674291 23250997

[15] Zhou J Guo P Hao X Sun X Feng H Chen Z. Neutrophil Extracellular Traps (NETs): A New Therapeutic Target for Neuroinflammation and Microthrombosis After Subarachnoid Hemorrhage? Transl Stroke Res. (2022) 14:443–5. doi: 10.1007/s12975-022-01039-y, PMID: 35689126 PMC9187359

[16] Chen GY Nuñez G. Sterile inflammation: sensing and reacting to damage. Nat Rev Immunol. (2010) 10:826–37. doi: 10.1038/nri2873, PMID: 21088683 PMC3114424

[17] Klune JR Dhupar R Cardinal J Billiar TR Tsung A. HMGB1: endogenous danger signaling. Mol Med. (2008) 14:476–84. doi: 10.2119/2008-00034.Klune, PMID: 18431461 PMC2323334

[18] Han DSC Lo YMD. The Nexus of cfDNA and Nuclease Biology. Trends Genet. (2021) 37:758–70. doi: 10.1016/j.tig.2021.04.005, PMID: 34006390

[19] Hakkim A Fürnrohr BG Amann K Laube B Abed UA Brinkmann V. Impairment of neutrophil extracellular trap degradation is associated with lupus nephritis. Proc Natl Acad Sci USA. (2010) 107:9813–8. doi: 10.1073/pnas.0909927107, PMID: 20439745 PMC2906830

[20] Grosse GM Blume N Abu-Fares O Götz F Ernst J Leotescu A. Endogenous Deoxyribonuclease Activity and Cell-Free Deoxyribonucleic Acid in Acute Ischemic Stroke: A Cohort Study. Stroke. (2022) 53:1235–44. doi: 10.1161/STROKEAHA.121.036299, PMID: 34991335

[21] Hao X Zeng Z Liang L Feng Z Li W Xiong B. The Role of Neutrophil Extracellular Traps in Early Microthrombosis and Brain Injury After Subarachnoid Hemorrhage in Mice. Transl Stroke Res. (2022) 14:752–65. doi: 10.1007/s12975-022-01074-9, PMID: 35962915 PMC9375080

[22] Zeng H Fu X Cai J Sun C Yu M Peng Y. Neutrophil Extracellular Traps may be a Potential Target for Treating Early Brain Injury in Subarachnoid Hemorrhage. Transl Stroke Res. (2022) 13:112–31. doi: 10.1007/s12975-021-00909-1, PMID: 33852132

